# The relationship between hospital ethical climate and continuing education in nursing ethics

**DOI:** 10.1371/journal.pone.0269034

**Published:** 2022-07-21

**Authors:** Ayaka Okumoto, Satoko Yoneyama, Chiharu Miyata, Ayae Kinoshita

**Affiliations:** 1 Department of Human Health Sciences, Graduate School of Medicine, Kyoto University, Kyoto, Japan; 2 Department of Neuropsychiatry, Kanazawa Medical University, Ishikawa, Japan; 3 Course of nursing science, Mie University Graduate School of Medicine, Mie, Japan; Sreenidhi Institute of Science and Technology, INDIA

## Abstract

**Background:**

In recent years, there has been a growing interest in the importance of creating a healthy ethical climate. Although relationship with various factors and the ethical climate have been reported, understanding of the relationship between ethical education and ethical climate is limited.

**Aim:**

This study aims to investigate the relationship between ethical climate, personal characteristics, and continuing education for ethics.

**Methods:**

This study conducted a quantitative cross-sectional survey of 605 nurses in 3 teaching hospitals in Japan. Multiple-regression analysis was used to assess the relationship between ethical climate and demographic characteristics and continuing education. Further mean of ethical climate scores were compared between received continuing education and did not, using analysis of covariance adjusted for demographic variables.

**Findings:**

The ethical climate showed significant association with hospital, gender, specialty of the unit, experience of ethics education, in-service ethical training, and workshops/ academic conferences on nursing ethics. In multiple-regression analysis, attending in-service ethical training increased the mean of ethical climate score (p = 0.031) and workshops/ academic conferences decreased the mean score (p = 0.028). Adjusted-mean of ethical climate score of nurses who had in-service training was significantly higher than those who had not (p = 0.038), whereas adjusted-mean of it of nurses who had attended workshops/ academic conferences was significant lower (p = 0.033).

**Discussion:**

In-service training on ethics was associated with the positive ethical climate. Hospital should enhance ethical education.

**Conclusion:**

Ethical climate related to the nurses’ personal characteristics and continuing education. We propose that organizational support for ethical education may be effective in raising the ethical climate of the workplace.

## Introduction

In recent years, there has been a growing interest in the importance of creating a healthy and ethical work environment in clinical practice [1]. The ethical climate is considered an essential component of the organizational climate and is defined as shared perceptions of what is ethically correct behavior and ways to handle ethical issues [2]. Ethical climate refers to an environment in the organization that an individual perceives to influence and set the standard for employee behavior [3].

To measure ethical climate objectively, researchers have developed various theories and measurements. In this study, the scale developed by Olson [4], the Hospital Ethical Climate Survey (HECS), was used to measure the ethical climate of hospitals. Using instruments, it has been argued that the ethical climate may influence nurses’ well-being in the workplace and the quality of patient care provision, making it a significant factor in the nursing environment [5]. Various studies show that an ethical climate may be associated with or affect moral distress [6–9], job satisfaction [10, 11] turnover intentions and medical error experience [12], satisfaction with the quality of care and collaboration [7], ethical leadership [11], and moral sensitivity [13, 14]. Additionally, research is being conducted to clarify the relationship between ethical climate and demographic factors such as age, gender, nurses’ education degree, material status, years of experience, employment status, job position, education degree, training related to ethics, type of work (daytime or shift), weekly work time, the average number of patients cared for, working conditions, etc. [11, 12, 15–18].

The ethical climate is the pervasive moral atmosphere of a social system, characterized by the shared perceptions of right and wrong, as well as common assumptions about how moral concerns should be addressed [19]. Therefore, investigating the ethical climate of hospitals contributes to enhancing the work environment and quality of care in the context of nursing [18]. The ethical climate is the shared perception formed in the nurses’ workplace. According to our previous study, nurses’ perceptions of bed alarm system and physical restraint were related to the hospital they belong to, years of experience and education [20]. Ethics education is a method for preventing or addressing moral distress [21] and has a positive influence on moral confidence, moral action [22], ethical decision making and ethical behavior [5]. The nurses and social workers with ethics education use ethics resources more often [22]. In this way, ethics education is an important factor for nurses’ moral perception and action. Therefore, we created a hypothesis that ethical education might change the perception of the ethical climate for nurses.

According to previous studies, there is no relationship between nurses’ education degree and ethical climate score [11, 12, 15, 16, 23, 24]. However, continuing education includes post-license education; thus, it may be associated with nurses’ perception of ethical climate. Grady posited access to continuing education as an indicator of the organization’s ethical climate and support for ethics [22]. Actions for promoting a positive ethical climate include providing support for the continuing education on nursing ethics [12]. In the research regarding continuing education, Borhani et al. stated that the history of attendance to ethical workshops was related to a more positive evaluation of the ethical climate at the workplace [23], while other researchers insisted that there was no statistical difference between HECS and receiving training related to ethics [11] or ethics workshops [17]. The study of the employees in the executive branch of the U.S. federal government has shown that ethics training had a consistently positive influence on employee perception of ethical climate [25]. Moreover, in McDaniel’s intervention study of critical care, the ethical environment score of nurses who received the ethics educational program was higher than that of nurses who had not [26].

Although these earlier studies investigated the relationship of several factors with ethical climate using the t-test, ANOVA, or single-liner model [11, 17, 24], adjusting for factors associated with ethical climate may yield different results. Therefore, in this report, we would like to more strictly focus on the relationship between nursing ethics education and ethical climate using multiple regression analysis, then elucidate the detailed educational contents that might influence nurses’ perception of ethical climate. In Japan, the main types of continuing education for registered nurses (RNs) and midwives are in-service training in the workplace and workshops/ academic conferences. We have, hence, focused on these types of ethics education.

## Methods

### Design, sample, and settings

This study implemented a quantitative cross-sectional survey. The instrument used was a self-administered questionnaire. To the request for cooperation in the Kansai region, three teaching hospitals agreed to participate in this survey. These hospitals are teaching hospitals / academic medical centers that are large, multi-unit, acute-care general hospitals. They are in charge of assisting in the training of undergraduate nursing students and newly graduated nurses. Since the main purpose of this study was to explore the relationship between ethics education and ethical climate, we selected these teaching hospitals where the nursing staff is considered to have a high interest in education.

The questionnaires were delivered to the hospitals directly or through the mail, who then distributed them among the nurses who directly cared for patients. Upon completion, the questionnaires were collected by the researchers from the hospitals or mailed directly to the researcher by the nurses themselves. The survey was conducted from December 2019 to January 2020, with a population of 2168 nurses working in 12 units at three teaching hospitals. While 2168 questionnaires were distributed, 770 responses were received, returning a rate of 35.5%. Out of the total responses, 165 having missing data for J-HECS26 were excluded from the analyses. The sample size was considered sufficient to meet 351 nurses, which is calculated by the Cochran formula [27].

### Measurement tools

Among the various scales of evaluation of ethical climate, the HECS is used most to measure the ethical climate of the nursing environment [5]. The HECS was developed by Olson [4] in the U.S. to measure how nurses perceive the ethical climate of their workplace through their perspective of relationships with peers, patients, managers, hospital, and physicians. It consists of 26 items and five subscales: peers (4 items), patients (4 items), managers (6 items), hospital (6 items), and physicians (6 items). The survey was designed in a Likert-type format with all items being positive and answers rated from 1 (almost never true) to 5 (almost always true). The HECS has been examined for validity and reliability and is widely used in various countries [12, 13, 16, 28–31]. In Olson’s study [4], the total value of Cronbach’s alpha coefficient was found to be 0.91 and the values of the five subscales were as follows: peers– 0.73, patients– 0.68, managers– 0.81, hospital– 0.92, and physicians– 0.77. To focus on the ethical atmosphere in hospitals, we chose HECS to measure nurses’ perceptions of ethical climate. In Japan, there are two types of translations of HECS, namely, J-HECS26 and J-HECS. The former, which we translated into Japanese, consists of all the 26 items in the original version, while the latter consists of 18 items, whose reliability and validity were confirmed in the study targeting the medium-sized hospitals with 180–500 beds [32]. The reliability and validity of J-HECS26 were verified in the study conducted in large teaching hospitals with 600–1100 beds [33]. The Cronbach’s alpha coefficient of J-HECS26 in this study was 0.94 and their subscale are 0.67–0.95, which are equivalent to the original HECS in the previous literature [33].

### Sociodemographic characteristics

The questionnaire in the current study also encompassed the following demographic variables: age, gender, years of experience, marital status, education degree, the hospital nurses work at, license, position, the specialty of the unit they belong to, the experience of ethics education, and the opportunity of attending ethical training in and out of the hospitals (in-service training in the workplace, workshops / academic conferences on nursing ethics).

### Regarding ethics education

In this research, we divided ethical education into two types; in-service training and the workshops/ academic conference. The in-service training on ethics in the workplace includes the ethics training sponsored by the hospital’s Nursing Department and the study session on ethics held in the hospital ward. This type of training is intended to meet the actual needs of the workplace. The workshops/ academic conferences are held several times a year or annually by the nurses’ associations or organizations. In the workshops/ academic conferences, researchers give research presentations or attend lectures on advanced studies for carrier development and continuous education. They are often held for academic purposes and the topics are usually general ethical problems.

### Ethical consideration

Ethical approval was obtained from the Research Ethics Committee of the Kyoto University (R0850). Each questionnaire had a cover letter explaining the purpose of the research. The identity of the participants was kept anonymous, and the return of the completed questionnaires was considered as evidence of informed consent for participation in the study. Each questionnaire was assigned a code number that helped mask the individual identities, and the responses from the nurses were kept confidential.

### Statistical analysis

To ensure the collection and analysis of data without compromising confidentiality, each questionnaire was treated anonymously, and each participant was indicated by a code number. The sample data were analyzed using the SPSS 24.0 J software for Windows (IBM Corporation). The mean and standard deviation for age and years of experience, and J-HECS26 and sub-scale items were calculated. As for the correlation between ethical climate and age, years of experience, a Pearson correlation was calculated. The means of J-HECS26 total score for the demographic variables of gender, marital status, education degree, hospital, unit (surgery, internal medicine, mixed surgery/internal medicine, psychiatry, obstetric, pediatrics, operating room, emergency department, stroke care unit (SCU), intensive care unit (ICU)/ coronary care unit (CCU), neonatal intensive care unit (NICU)), license (Midwife, Advanced licensed nurse), job position, and continuing education were evaluated using t-test, one-way analysis of variance (ANOVA), and Kruskal-Wallis test. Multivariate linear regression models were developed to analyze the relationship between the J-HECS26 and the independent variables: demographic variables and continuing education. Model 1 included the years of experience (continues), gender, job position, license (dummy variable), hospital (dummy variable), and 11 units (dummy variable). Model 2 included all the variables in Model 1, along with in-service training on ethics in the workplace. Model 3 included Model 2 variables plus workshops/ academic conferences on nursing ethics. The means of J-HECS26 score were compared between the data for nurses who have attended and those who did not attend in-service training in the workplace on ethics, and workshops/ academic conferences on nursing ethics, using analysis of covariance adjusted for year of experience (continues), gender, job position, license (dummy variable), hospital (dummy variable), and 11 units (dummy variable). The level of significance chosen for this study was .05.

## Results

### Demographics

The demographic data are shown in Table 1. Of the total 605 nurses, 561 (92.7%) who answered the questionnaire were females and 44 were males (7.3%) with a mean age of 31.4 ± 8.3 years. The mean number of years of their experience was 9.3 ± 8.0. Among the sample population, 167 (27.7%) had a nursing diploma, 369 (61.0%) had a bachelor’s degree, and 20 (3.3%) had a master’s / doctoral degree, while 48 (7.9%) were junior college graduates. Concerning ethics educational opportunities, 430 (71.1%) nurses had ethics education, while 175 (28.9%) nurses did not. Regarding the type pf education, 393 (65.0%) nurses attended in-service training at the workplace and 122 (20.2%) attended workshops/ academic conferences (Table 1).

**Table 1:** Demographic characteristics (n = 605).

Variables		n	(%)	Mean	SD
Age (year)				31.4	8.3
Years of experience (year)				9.3	8.0
Gender					
	Female	561	(92.7)		
	Male	44	(7.3)		
Education degree					
	Nursing diploma	167	(27.7)		
	Junior college	48	(7.9)		
	Bachelor’s	369	(61.0)		
	Master’s/doctoral	20	(3.3)		
	No answer	1	(0.2)		
Hospital					
	1	410	(67.7)		
	2	130	(21.6)		
	3	65	(10.7)		
License					
	Resisted Nurse	561	(92.7)		
	Midwife	29	(4.8)		
	Advanced Licensed Nurse	14	(2.4)		
	No answer	1	(0.1)		
Position					
	Staff	531	(87.8)		
	Manager	74	(12.2)		
Unit					
	Surgery	201	(33.3)		
	Internal medicine	174	(28.8)		
	Mixed surgery and internal medicine	69	(11.4)		
	ICU/CCU	35	(5.8)		
	NICU	28	(4.6)		
	Obstetric	22	(3.6)		
	Pediatric	19	(3.1)		
	Operating room	16	(2.6)		
	Psychiatry	15	(2.5)		
	Emergency department	12	(2.0)		
	SCU	7	(1.2)		
	Others	7	(1.0)		
Experience of ethical education					
	Yes	430	(71.1)		
	No	175	(28.9)		
In-service training in workplace					
	Yes	393	(65.0)		
	No	212	(35.0)		
Workshops/ Academic conferences on nursing ethics					
	Yes	122	(20.2)		
	No	483	(79.8)		

*Note;* SD = standard deviation

### Nurses’ level of ethical climate

The mean score of J-HECS26 was 94.8 ± 14.8. The mean scores for the five sub-scales of the HECS, namely, managers, hospital, physicians, peers, and patients were 22.5 ± 5.9, 21.0 ± 3.6, 19.8 ± 4.2, 16.3 ± 2.5, and 15.2 ± 2.0, respectively (Table 2).

**Table 2:** J-HECS26 score (n = 605).

	Mean	SD	Min	Max
Total score of J-HECS26	94.8	14.8	47	130
Peers (4 items)	16.3	2.5	8	20
Patients (4 items)	15.2	2.0	8	20
Managers (6 items)	22.5	5.9	6	30
Hospital (6 items)	21.0	3.6	10	30
Physicians (6 items)	19.8	4.2	6	30

### Demographic variables and J-HECS26

An independent sample correlation, t-test, and ANOVA were conducted to examine whether there is any relationship between J-HECS26 and the demographic variables. There was a weak negative correlation between J-HECS26 and age (r = -0.130, p = 0.002), years of experience (r = -0.136, p = 0.001). In the results of the t-test, one-way ANOVA, and Kruskal-Wallis test, a significant difference was found between J-HECS26 and demographic variables of the hospital (p = 0.001) and units (p < 0.001); notably, the score of nurses working in operating rooms was the lowest (p < 0.001) (Table 3). Female nurses scored higher than males (p = 0.042) and the nurses who have had ethics education got a higher score than those who have not had any (p = 0.035). Regarding continuing education, nurses who have attended in-service training at the workplace scored higher in J-HECS26 than non-participants (p < 0.001). Further, the nurses who had attended workshops/ academic conferences on nursing ethics scored lower than those who had not (p = 0.023). The difference between J-HECS26 and demographic variables of marital status, education degree, license, and position were not statistically significant.

**Table 3:** Association between demographic variables and J-HECS26 total score (n = 605).

		n	Mean	SD	Min	Max	p value
Gender^†^							0.042*
	Female	561	95.1	14.8	47	130	
	Male	44	90.4	14.6	62	128	
Hospital^‡^							0.001*
	1	410	93.4	14.9	47	130	
	2	130	96.8	14.1	51	127	
	3	65	99.5	14.6	62	130	
License^‡^							0.174
	Registered Nurse	562	94.7	14.8	47	130	
	Midwife	29	98.6	14.2	71	127	
	Advanced Licensed Nurse	14	89.8	16.8	60	121	
Position^†^							0.558
	Manager	74	95.7	13.4	50	121	
	Staff	531	94.6	15.0	47	130	
Unit^§^ (n = 604)							< 0.001*
	Surgery**	201	92.9	14.8	47	122	
	Internal medicine**	174	97.3	12.0	63	128	
	Mixed surgery and internal medicine**	69	99.0	13.4	64	126	
	Psychiatry**	15	103.9	14.1	76	130	
	Pediatric**	19	95.8	19.2	53	127	
	Obstetric**	22	102.9	12.8	75	127	
	Emergency department**	12	98.6	15.7	72	124	
	Operating room	16	73.0	11.8	50	95	
	ICU/CCU	35	88.1	13.9	61	113	
	SCU	7	91.0	6.8	77	98	
	SCU NICU**	28	92.1	19.5	53	130	
	Others	7	87.7	14.5	71	116	
Experience of ethics education^†^							0.035*
	Yes	430	95.6	14.2	50	130	
	No	175	92.8	16.0	47	130	
In-service training in workplace on ethics^†^(n = 604)							< 0.001*
	Yes	393	96.3	14.0	50	130	
	No	211	91.8	15.8	47	130	
Workshops/ Academic conferences on nursing ethics^†^ (n = 604)							0.023*
	Yes	122	92.0	14.4	50	127	
	No	482	95.4	14.9	47	130	

Notes

^†^ student’ t-test

^‡^ one-way ANOVA: one-way analysis of variance

^§^ Kruskal-Wallis test; SD = standard deviation.

* p< 0.05

** Multiple comparison test p< 0.05 versus operating room

Multiple regression analysis was used to assess the association between demographic variables and J-HECS26 (Table 4). The mean J-HECS26 score showed significant increase in manager nurses (p = 0.033) and nurses in the psychiatry unit (p = 0.007) obstetrics unit (p = 0.014), and emergency department (p = 0.031) and showed significant decrease in nurses with less experience *(p =* 0.002), male nurses (p = 0.020), and nurses in the operating room (p = 0.004).

**Table 4:** Results of multiple regression analysis with J-HECS26 (n = 604).

	Model 1^†^			Model 2^‡^			Model 3^§^		
	Coefficient	t	p value	Coefficient	t	p value	Coefficient	t	p value
Years of experience	-0.150	-3.1	0.002*	-0.151	-3.1	0.002*	-0.132	-2.7	0.007*
Gender	-0.091	-2.3	0.020*	-0.090	-2.3	0.022*	-0.089	-2.3	0.022*
Manager	0.105	2.1	0.033*	0.092	1.9	0.064	0.110	2.2	0.029*
Midwife	-0.073	-1.0	0.329	-0.076	-1.0	0.311	-0.072	-1.0	0.336
Advanced license nurse	-0.042	-1.0	0.298	-0.040	-1.0	0.327	-0.030	-0.7	0.465
Hospital 1	-0.152	-2.4	0.017*	-0.127	-2.0	0.049*	-0.116	-1.8	0.073
Hospital 2	-0.060	-1.0	0.339	-0.055	-0.9	0.383	-0.036	-0.6	0.569
Surgery	0.067	0.5	0.617	0.080	0.6	0.549	0.080	0.6	0.548
Internal medicine	0.210	1.6	0.110	0.220	1.7	0.093	0.223	1.7	0.088
Mixed surgery and internal medicine	0.143	1.5	0.138	0.151	1.6	0.117	0.155	1.6	0.106
Psychiatry	0.156	2.7	0.007*	0.159	2.8	0.006*	0.158	2.8	0.006*
Pediatric	0.066	1.1	0.284	0.060	1.0	0.331	0.063	1.0	0.303
Obstetrics	0.213	2.5	0.014*	0.214	2.5	0.013*	0.217	2.5	0.012*
Emergency department	0.106	2.2	0.031*	0.109	2.2	0.026*	0.107	2.2	0.029*
Operating room	-0.170	-2.9	0.004*	-0.162	-2.8	0.006*	-0.160	-2.7	0.007*
ICU/CCU	-0.026	-0.3	0.734	-0.023	-0.3	0.761	-0.019	-0.3	0.795
SCU	0.000	0.0	0.996	0.005	0.1	0.913	0.006	0.1	0.906
NICU	0.022	0.3	0.762	0.029	0.4	0.681	0.031	0.4	0.661
In-service training in workplace on ethics				0.088	2.2	0.031*	0.089	2.2	0.028*
Workshops/ Academic conferences on nursing ethics							-0.092	-2.2	0.028*
R^2^	0.156			0.164			0.171		

Notes

^†^ Model1: Un-adjusted

^‡^ Model2: Adjusted for Model 1 covariates

^§^ Model3: Adjusted for Model 2 covariates.

* p< 0.05

### Continuing education

Further multiple-regression analysis was conducted to assess the association between continuing education and J-HECS26 scores (Table 4). In Model 2, the mean J-HECS26 score showed a significant increase in nurses who had attended in-service training at the workplace (p = 0.031). In Model 3, the mean J-HECS26 score significantly decreased in nurses who had attended workshops/ academic conferences (p = 0.028).

Table 5 shows the multivariate-adjusted mean of the J-HECS26 score. The nurses who had attended in-service training at the workplace scored 2.63 points, which was significantly higher than those who had not attended any (p = 0.038, 95% CI: 0.15, 5.11). Nurses who had attended workshops/ academic conferences scored 3.30 points lower than those who did not (p = 0.033, 95% CI: - 6.33, - 0.28).

**Table 5:** Difference of multivariate-adjusted mean values of J-HECS26 (n = 604).

	(Yes)			(No)			(Yes)- (No)			
	Mean	95% CI		Mean	95% CI		Mean	95% CI		p value
In-service training in workplace on ethics	95.7	94.3	97.0	93.0	91.0	95.0	2.63	0.15	5.11	0.038*
Workshops/ Academic conferences on nursing ethics	92.1	89.4	94.8	95.4	94.1	96.7	-3.30	-6.33	-0.28	0.033*

*Notes*: Adjusted for year of experience (continues), gender, hospital (dummy variables), manager, unit (surgery, internal medicine, mixed surgery and internal medicine, psychiatry, pediatric, obstetric, emergency department, operation, ICU/CCU, SCU, NICU: dummy variables), license (midwife, advanced license nurse: dummy variables); CI = confidence interval.

* p< 0.05

## Discussion

The purpose of this study was to ascertain the factors that might affect the ethical climate by using J-HECS26, which we first translated into Japanese before distributing it among the participants. We focused on the demographic data and education since the detailed influence of education on HECS has not been fully reported so far.

### Nurses’ level of ethical climate

The present study indicated that the mean score of J-HECS26 and sub-scales were 94.8. The mean score of J-HECS26 in this study was more than that in Khalesi [34] (71.5) and Shafipour, Yaghobian, Shafipour, and Heidari [35] (91.8), whereas it was less than that in Bansal [16] (116.7) and Tehranineshat [36] (100.1). All these studies were conducted in teaching hospitals, as was done in our study. It was found that nurses in this study perceived their ethical climate as relatively desirable.

### Demographic characteristics

There was a significant relationship between nurses’ demographic characteristics and their perception of ethical climate. Younger nurses and those with less experience have a more positive perception of ethical climate. Being a female was related to a more positive evaluation of the ethical climate of the hospitals where they were employed. Additionally, nurses’ perception of ethical climate was significantly related to the hospital and unit where they worked. There are several previous studies regarding factors influencing the HECS, although some are rather controversial. Some studies reported that age, gender, years of experience, and degree of education (i.e., nursing diploma /associate /bachelor’s /master’s degree) had no significant impact on the HECS scores [16, 18, 37], whereas other studies showed that age [12, 15], gender [11, 24], job position [12], years of experience [11, 12, 13, 15] and education degree (bachelor’s /master’s /doctoral degree) did have a statistically significant effect on the HECS [17].

Concerning years of experience, inexperienced nurses were reported to perceive ethical climate optimistically [13, 15], which is consistent with our results in this current study. Concerning gender, similar to our findings, Ozden et al. reported a significant difference between the gender of nurses and ethical climate perceptions, claiming that their result reflected the general situation of the majority of participants in the survey being female, as Turkish nurses were predominantly female [11]. In our study, 92% of the participants were female, which is similar to the overall proportion of nurses in Japan [38]. There are several reports that male nurses feel more difficulty with working in a female-centered workplace as compared to female nurses in terms of interacting with female patients and female nurses and anxiety regarding their future career development [39]. Therefore, male nurses might recognize their perception of ethical climate more negatively than female nurses.

Further, the t-test results showed that there was no significant statistical difference between the perception of the ethical climate of manager nurses and staff nurses. However, adjusting for variables such as years of experience and gender, managers have significantly higher perceptions of ethical climate. In previous literature, Hwang and Park reported that manager nurses perceive ethical climate more positively than staff nurses [12]. Our study is in accordance with their study, demonstrating a similar result even after adjusting for years of experience. These studies, including ours, indicate that the managers might generally commit to the ethical procedures and policies of the organization and collaborate with doctors and colleagues on ethical issues.

Regarding the hospital and specialty of the unit, previous studies suggested that the staff in different hospitals had different perceptions of the ethical climate [11, 12, 15]. Ozden et al.’s study [11], which was conducted in a university-attached hospital and an education and research hospital, similar to our study, reported a significant difference in the ethical climate among hospitals. Our study supports their report.

On the other hand, a review of the related studies presents conflicting results regarding the relationships between the unit and nurses’ perceptions of ethical climate. Hwang and Park [12] argued that the HECS scores were not related to the specialties of the units, while Ozden et al. [11] and Lemmenes et al. [40] confirmed that they were indeed related to this variable. This inconsistency might be relevant to the differences between the researched hospitals. Our results showed that the ethical climate differs among the units; in particular, nurses in the operating room showed significantly lower mean scores of J-HECS26. Rosenstein and O’Daniel noted that the operating room is physically small, there is a strong interdependence on effective team functioning, and working in this unit is highly stressful [41]. According to Sonoda, Onozuka, and Hagihara, operating room nurses have also been reported to be mentally stressed not only by doctors but also by colleagues [42]. Circulating nurses in the operation room may feel frustrated by the scrub nurses [42], presumably leading to a negative perception of the workplace. The low mean score of nurses in the operating room in our study might reflect a negative relationship with these colleagues in the operation room or at the workplace.

### Continuing education

Other than the aforementioned demographic characteristics, we found that ethics education may have influenced the nurses’ perception of the ethical climate. This study showed that nurses who had attended in-service training at the workplace perceived their ethical climate positively; however, the nurses who had participated in the workshops/ academic conferences found their hospital ethical climate to be negative, which differed from our expectations. This result was verified through multiple regression analysis and analysis of covariance.

Previous studies showed conflicting results regarding the effect of ethics education on the HECS scores. While some studies showed no relationship between ethical climate and attendance of ethical workshop/ training [11, 17], Borhani et al. reported a positive effect of education on ethical climate, indicating that the nurses who participated in an ethics workshop perceived their ethical climate as more positive [23]. These differences might depend on the style of education or programs. Previous literature pointed out that interactive workshops, rather than uni-directional lectures, enlighten nurses more [43]. An ethical climate represents “shared perceptions” of organizational practices related to ethical decision-making [3]. In-service training in the workplace gives nurses a consistent awareness of the ethics practiced in the particular hospital where they are employed. As a result, nurses who attend in-service training may be more positive about the ethical climate of their workplace than those who did not.

On the other hand, nurses who participated in off-the-job workshops/ academic conferences perceive their climate more negatively than those who did not participate. At workshops/ academic conferences, nurses acquire the most advanced ethical knowledge, values, and perception. However, it is unclear whether this knowledge will be properly utilized in the nursing practice of their workplace. In fact, one study shows that off-the-job training may not directly apply to the workplace of nurses [44]. Therefore, what they have learned there may not have been relevant to their practical situation; thus, they may not have been able to apply what they have learned or share it with their colleagues. These frustrations might explain the negative relationship between ethical climate and the out-of-hospital ethical training found in this study. Therefore, what they have learned at off-the-job workshops/ conferences should be reviewed and incorporated into their workplace with caution to ensure that their acquired knowledge finds more use in practice.

Additionally, having education outside the hospital is not enough to enhance the workplace’s ethical climate. Ethical climate is nurses’ shared perception. Even if a nurse adapts the latest knowledge at an academic conference or out-of-hospital educational opportunities, it may be difficult to put it into practice if it is not properly shared with colleagues. On the contrary, the same knowledge is shared by the nurses of the same hospital at in-hospital training, which can transform the acquired knowledge into practical use. Ulrich also states that receiving ethical education alone may leave nurses dissatisfied unless it is properly practiced in the workplace [45]. This supports our result that in-hospital training is more related to the positive ethical climate.

Moreover, the motivation for nurses to receive continuing professional education is to improve knowledge, patient care delivery, and professional relationships [46]. There is a possibility that the nurses who had attended the out-of-hospital education had negative feelings about their current workplace, involving concerns related to ethical issues and seeking solutions. Accordingly, the nurses who had attended the out-of-hospital ethical education might have a negative perception of the ethical climate of their workplace in this study. Resolving the frustrations and concerns of these nurses may lead to reducing their moral distress and preventing their intention to leave the organization. Simultaneously, the HECS may be useful in identifying the nurses who need support from the workplace to make the best of their eagerness to improve the ethical atmosphere.

In summary, our study has new implications for the importance of the provision of ethics education and clarifies that offering in-service ethical training that meets the actual needs of their workplace may provide nurses a shared perception of ethical climate. Our result demonstrates that not only individual efforts but also organizational efforts are essential for improving the ethical climate of a workplace. Further research should elucidate the kind of education and organizational support that is effective in enhancing the ethical climate.

### Strength and limitations

The strength of this study is that it is the first to use J-HECS26 (translated into Japanese), with all 26 items of the original HECS. Further, our study clearly demonstrated a relationship between demographic characteristics and ethics education by using multiple regression analysis. However, our study did not clarify the details of ethics education (i.e., timing, frequency, and content). Moreover, since educational support differs depending on the hospital, a selection bias may have occurred. Therefore, future studies must analyze hospitals of other sizes and that offer different services in order to clarify the details of education. Ethical climate has been reported to be related to various other factors such as ethical stress or job satisfaction. Thus, another limitation is that these factors were not measured in our questionnaire.

## Conclusion

This study examined nurses’ perceptions of the ethical climate in Japanese teaching hospitals. Nurses’ perception of ethical climate varied based on individual characteristics such as years of experience, gender, job position, hospital, and specialty of the unit. We also demonstrated that in-service training, adjusted for the workplace, is effective in raising employee perception of the ethical climate. Lastly, off-the-job workshops/ academic conferences are not directly related to enhancing the ethical climate and, hence, should be implemented carefully.

## Supporting information

S1 Dataset (XLSX)
